# Predicted Potential for Aquatic Exposure Effects of Per- and Polyfluorinated Alkyl Substances (PFAS) in Pennsylvania’s Statewide Network of Streams

**DOI:** 10.3390/toxics12120921

**Published:** 2024-12-19

**Authors:** Sara E. Breitmeyer, Amy M. Williams, Matthew D. Conlon, Timothy A. Wertz, Brian C. Heflin, Dustin R. Shull, Joseph W. Duris

**Affiliations:** 1 Pennsylvania Water Science Center, U.S. Geological Survey, New Cumberland, PA 17070, USA; mconlon@usgs.gov (M.D.C.); jwduris@usgs.gov (J.W.D.); 2 Bureau of Clean Water, Pennsylvania Department of Environmental Protection, Harrisburg, PA 17101, USA; amywilli@pa.gov (A.M.W.); twertz@pa.gov (T.A.W.); dushull@pa.gov (D.R.S.); 3 Independent Researcher, Colorado Springs, CO 80906, USA

**Keywords:** PFAS, water quality, streams, PFAS aquatic exposure, machine learning, biotic sampling prioritization

## Abstract

Per- and polyfluoroalkyl substances (PFAS) are contaminants that can lead to adverse health effects in aquatic organisms, including reproductive toxicity and developmental abnormalities. To assess the ecological health risk of PFAS in Pennsylvania stream surface water, we conducted a comprehensive analysis that included both measured and predicted estimates. The potential combined exposure effects of 14 individual PFAS to aquatic biota were estimated using the sum of exposure-activity ratios (ΣEARs) in 280 streams. Additionally, machine learning techniques were utilized to predict potential PFAS exposure effects in unmonitored stream reaches, considering factors such as land use, climate, and geology. Leveraging a tailored convolutional neural network (CNN), a validation accuracy of 78% was achieved, directly outperforming traditional methods that were also used, such as logistic regression and gradient boosting (accuracies of ~65%). Feature importance analysis highlighted key variables that contributed to the CNN’s predictive power. The most influential features highlighted the complex interplay of anthropogenic and environmental factors contributing to PFAS contamination in surface waters. Industrial and urban land cover, rainfall intensity, underlying geology, agricultural factors, and their interactions emerged as key determinants. These findings may help to inform biotic sampling strategies, water quality monitoring efforts, and policy decisions aimed to mitigate the ecological impacts of PFAS in surface waters.

## 1. Introduction

Per- and polyfluoroalkyl substances (PFAS) are a growing environmental concern due to their widespread presence in aquatic ecosystems and potential to harm both human health and aquatic life. PFAS can accumulate in fish tissues and often exceed safe consumption levels for humans (De Silva et al. [1]; Sunderland et al. [2]). This contamination has led to “Do-Not-Eat” advisories being issued to protect public health around the globe. For example, the state of Pennsylvania issued a 2024 health advisory to not eat more than eight ounces per week of recreationally caught sport fish (Pennsylvania Fish and Boat Commission [3]). Furthermore, a primary human exposure pathway for PFAS is through drinking water, often sourced from environmental surface waters. In 2024, the U.S. Environmental Protection Agency (EPA) finalized regulations of enforceable limits for six PFAS in public supply drinking water (U.S. Environmental Protection Agency [4]).

The risks of PFAS contamination in environmental waters extends beyond human consumption, with PFAS exceeding EPA aquatic life criteria and environmental quality standards in numerous instances (Ruffle et al. [5]). In aquatic biota, metabolic and reproductive endpoints are disproportionately studied compared to effects on vision, oxidative toxicity, development, endocrine, and immunological modifications. Observed effects of PFAS exposure to fish (typically from perfluorooctanoic acid (PFOA) and perfluorooctanesulfonic acid (PFOS)) have included adverse changes in body size, swimming activity, and liver size (Banyoi et al. [6]). Furthermore, exposure to perfluorooctanesulfonamide (PFOSA), PFOS, and perfluorohexanesulfonic acid (PFHxS) at environmentally relevant concentrations leads to increased fish mortality rates. Exposure to individual PFAS results in limited effects on whole organism development but a higher prevalence of developmental defects in the body axis, swim bladder, pigmentation, and eyes (Hamed et al. [7]).

To assess the ecological health risks of environmental PFAS contamination, state and national governments are beginning to establish monitoring networks. These networks often require the collection of biological tissue and surface water samples for PFAS analysis and aim to measure varying levels of PFAS exposure, from minimal to severe. However, such monitoring can be costly. To inform these sampling efforts, researchers have estimated the potential effects of organic contaminants on biological life by comparing contaminant concentrations to environmental health benchmarks, toxicity data, and in vitro high-throughput screening data (e.g., EPA ToxCast) (Olker et al. [8]; Stackpoole et al. [9]; Shoda et al. [10]; Dix et al. [11]; Bradley et al. [12]; Corsi et al. [13]). ToxCast provides a vast dataset of chemical-biological interactions, featuring hundreds of assays on thousands of chemicals and assesses their biological relevance at measured concentrations (DeCicco et al. [14]; U.S. Environmental Protection Agency [15]).

Recent studies have underscored the effectiveness of machine learning (ML) models in predicting and analyzing PFAS contamination in environmental water and fish tissue, with a focus on key performance metrics. For instance, McMahon et al. [16] employed boosted regression tree models, achieving an accuracy of 84% in predicting PFAS detections in groundwater. Similarly, Dong et al. [17] utilized multilabel semisupervised machine learning techniques to predict 35 PFAS in California groundwater, attaining an area under the curve and receiver operating characteristic (AUC-ROC) ranging from 73% to 100% for individual PFAS and further emphasizing ML’s potential in environmental monitoring. DeLuca et al. [18] followed a similar approach, utilizing random forest models to forecast PFAS contamination in fish tissue with accuracy ranging from 71% to 82%. Recent Quantitative Structure-Property Relationship models have also achieved high predictive accuracy (e.g., 84% for 13 PFAS) (Kowalska et al. [19]).

Furthermore, ensemble ML methods, which combine multiple models to improve predictive accuracy, have been increasingly used to study PFAS contamination. Studies have shown that combining Gradient Boosting, Random Forest, and Neural Network models can outperform individual models in PFAS prediction (Khaki and Wang [20]). These methods can also be applied to broader ecological modeling, highlighting their robustness and potential for future environmental prediction tasks. Recent research has explored the integration of deep learning techniques, such as CNNs and recurrent neural networks (RNNs), into ecological and water quality prediction frameworks (Pyo et al. [21]; Gandhimathi et al. [22]; Pu et al. [23]; Limbu et al. [24]).

In PFAS prediction frameworks, deep learning approaches have demonstrated promise in capturing complex, non-linear relationships within environmental data, improving both prediction accuracy and generalizability across diverse geographical regions (Khaki and Wang [20]; Limbu et al. [24]). For example, a project led by researchers at Argonne National Laboratory utilized CNNs to estimate the toxicity and environmental exposure risks of PFAS compounds (Feinstein et al. [25]). The continued refinement of these models, along with the exploration of new ML methodologies, is essential for addressing the challenges posed by PFAS contamination, ensuring that predictive tools are both reliable and adaptable to varying environmental contexts.

In this study, we introduce a robust ML approach to assess the potential biological exposure effect of PFAS contamination to aquatic biota in stream surface water. This approach aims to inform targeted monitoring, potentially reducing associated costs. We compiled surface water PFAS concentrations analyzed from 280 streams that were sampled by various agencies. These concentrations were used to estimate the combined ToxCast exposure effects of 14 PFAS on aquatic biota (referred to hereafter as PFAS bioeffect potential), which were then utilized to train ML models for predicting the PFAS bioeffect potential across the entire stream network of Pennsylvania (PA). Alongside developing a sampling prioritization scheme, we evaluated four classical ML models and a tailored CNN, with the CNN demonstrating the best results, confirmed through model validation.

## 2. Materials and Methods

### 2.1. Data Preparation

#### 2.1.1. Stream Surface Water PFAS Concentrations

Stream surface water PFAS concentrations were retrieved from the EPA Enforcement and Compliance History Online (ECHO) PFAS Analytics Tool (U.S. Environmental Protection Agency [26]), U.S. Geological Survey (USGS) National Water Information System (NWIS) (U.S. Geological Survey [27]), and Sierra Club Moshannon Group (Roberts [28]). The PFAS concentrations from EPA ECHO were reported by the Delaware River Basin Commission and the USGS PA Water Science Center (PAWSC)/PA Department of Environmental Protection (PADEP) and those from the USGS NWIS were reported by the USGS New Jersey (NJ) Water Science Center (U.S. Environmental Protection Agency [26]; Breitmeyer et al. [29]; U.S. Geological Survey [27]). National Hydrography Dataset Plus (NHDPlus) v2.1 flowline ComIDs (i.e., a common identifier that uniquely identifies individual stream reaches; (McKay et al. [30])) were fetched and visually verified utilizing site geographic coordinate points. The combined data included PFAS concentrations for 280 stream reaches, with 19 compounds detected throughout all sites that were either sampled once or at multiple times. For each stream reach observation, detected compound concentrations were summed to total PFAS (ΣPFAS) concentration. To assess the potential for the maximum effect of ΣPFAS on biological life, for the 33% of stream reaches having PFAS samples collected at multiple times, the individual PFAS observations from the sampling date that had the maximum ΣPFAS concentration were kept for analysis. This conservative approach aligns with the principle of using worst-case scenarios that represent maximum PFAS exposures and a more cautious screening level to protect aquatic organisms.

#### 2.1.2. Exposure Activity Ratios

The R system (version 4.4.0; R Core Team [31]) and the USGS toxEval package (version 1.3.2) were utilized to assess the potential biological relevance of PFAS surface water concentrations that might be harmful to aquatic biota. The toxEval software compares measured concentrations to those that cause biological activity in EPA ToxCast assays (DeCicco et al. [14]; U.S. Environmental Protection Agency [15]). The assays, from ToxCast database version 3.5, utilize mainly vertebrate cell lines (e.g., DNA, proteins, receptors, and enzymes) to measure exposure response and thresholds of biological response, which range from endocrine disruption to neurological effects (U.S. Environmental Protection Agency [15]; Bradley et al. [12]). Of the 19 PFAS detected throughout study streams, 14 PFAS had Chemical Abstract Service Registry Number (CASRN) ToxCast matches, and 12 of the 14 had measurable effects within the range of detected PFAS concentrations. These 12 PFAS included perfluorohexanoic acid (PFHxA), PFOS, perfluorononanoic acid (PFNA), PFOA, perfluoroheptanoic acid (PFHpA), PFHxS, perfluorobutanoic acid (PFBA), perfluorobutanesulfonic acid (PFBS), perfluoroundecanoic acid (PFUnDA), perfluorodecanoic acid (PFDA), perfluoroheptanesulfonic acid (PFHpS), and PFOSA.

The ToxCast high-throughput assays generate concentration-response curves for each of the individual PFAS-endpoint pairings. These endpoints represent specific biological processes relevant to environmental hazard assessment in aqueous solution (Blackwell et al. [32]). For example, an individual exposure-activity ratio (EAR) greater than 1.0 indicates that the aqueous compound concentration is greater than the ToxCast assay endpoint concentration (DeCicco et al. [14]). The ToxCast assay endpoints utilized in this study were selected following methodology outlined in Corsi et al. [13]. This process involved an evaluation of data quality, examination of dose-assay response curves, and assessment of the reliability and quality of the endpoints for detecting both signal gains and losses. Several ToxCast assays excluded from analysis due to low quality dose-response curves based on anomalous values or lack of response are detailed in Table S2.

The ToxCast data analysis pipeline employs various summary metrics derived from chemical dose-assay response curves (Filer et al. [33]). For this research, the Activity Concentration at Cutoff (ACC) metric was chosen for comparison with water concentrations, aligning with previous studies (Blackwell et al. [32]; Fay et al. [34]; Corsi et al. [35]). A more comprehensive explanation of ACC derivation can be found in Judson et al. [36] and Filer et al. [33]. The minimum ACC value for each chemical was used as the final bioactivity concentration in the calculation of EARs.

For the individual detected PFAS that had concentrations high enough to result in a measurable EAR (>10^−6^), site-wise individual EARs were summed (ΣEAR) to represent the ΣPFAS concentration that induced a response in each ToxCast biological assay (Table S3). The ToxCast EAR offers a protective screening level that provides information about the potential sub-lethal effects to vertebrates (Bradley et al. [12]). However, limitations of the ToxCast EAR include the incomplete analytical coverage of all detected PFAS (e.g., 14 PFAS had ToxCast assays, but 5 other PFAS were detected that had no assays) and the poorly understood bioassay molecular-level effects to the organism level (Bradley et al. [12]). The authors acknowledge that these methods are used strictly as a screening and prioritization technique and that they must be validated with direct biological assays or biological health metrics collected in the field and/or laboratory (DeCicco et al. [14]).

#### 2.1.3. Geospatial Predictors

Geospatial predictors were gathered for NHDPlus v2.1 stream reach ComIDs (*n* = 111,735) within the Water Boundary Dataset v2.3.1 fourth-level watersheds (HUC4s) encompassing PA and NJ state boundaries (U.S. Geological Survey [37]). Predictors were gathered or derived from comprehensive national sources (U.S. Environmental Protection Agency [26]; Jones et al. [38]; Blodgett and Johnson [39]). To ensure that no potential predictors were overlooked and to create a straightforward reproducible workflow, a kitchen-sink approach was utilized to retain predictors. For temporal predictors (e.g., annual temperature and precipitation), the most recent predictor to sampling date was kept for analysis. Predictors included those representative of hydrology, climate, chemistry, geology, land cover, population infrastructure, water use, potential PFAS sources, and more (Table S4). To assess the influence of the direct drainage area on the PFAS biological effect potential and to capture potential PFAS sources close to the point of stream entry, we used predictors representative of the reach catchment of each site (i.e., the local scale).

Thirty-one predictors of potential PFAS sources were downloaded from the EPA ECHO website (U.S. Environmental Protection Agency [26]) as geospatial latitude and longitude points. To determine the reach catchment identity (NHD plus v2.1 FeatureID Gridcode) of the points, the intersect option was utlized in ArcPro (version 3.2.2). In each reach catchment, the points for each predictor (i.e., ECHO airports, ECHO electronics industry, etc. (Table S4)) were summed, divided by the respective reach catchment area to obtain densities, and matched to NHDPlus v2.1 ComIDs. Summaries of sinkholes in the reach catchments were computed utilizing the USGS xstrm local package (Wieferich et al. [40]) and a USGS geospatial layer of closed depression density in U.S. karst regions (Jones et al. [38]). Using the R system (R Core Team [31]) and nhdplusTools package (Blodgett and Johnson [39]), all of the other predictors were retrieved from Wieczorek et al. [41].

Subsequently, all predictors were normalized using the bestNormalize R package (version 1.9.1; Peterson and Cavanaugh [42]). The package provides a flexible solution for normalizing data, accommodating a wide range of distributions. By comparing the normality of different transformations using the Pearson P test and repeated cross-validation, bestNormalize identifies the most suitable transformation.

### 2.2. Study Area

The study area includes HUC4 watersheds in the Northeast U.S. and partially encompasses the Great Lakes, Ohio, and Mid-Atlantic watersheds. Aggregated ecoregions include the Northern and Western Allegheny Plateau, Erie Drift Plain, Ridge and Valley, Central and North Central Appalachians, Northern Piedmont, Northeastern Highlands, and Middle Atlantic Coastal Plain (U.S. Environmental Protection Agency [43]). The study area is roughly 50% forested, 20% agricultural, and 15% developed (Wieczorek et al. [41]). From 2016 through 2023, 280 non-tidally influenced streams having an observed median local watershed area of 3.3 km^2^ (range: 0.04–46.2 km^2^) were sampled to determine the concentrations of a suite of PFAS, which were utilized to derive the site-specific ΣEAR (detailed in Section 2.1.2). Streams varied in size, ranging from small first-order to larger eighth-order streams, with a median Strahler stream order of four.

**Figure 1:**
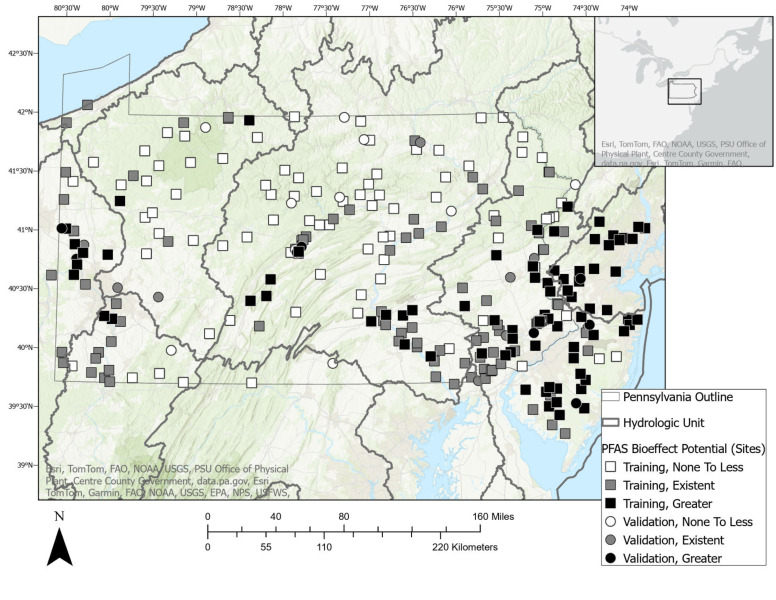
Map of the PFAS bioeffect potential in 280 stream sites in watersheds of Pennsylvania and New Jersey, U.S. Machine learning training sites are represented by squares and validation sites are represented as circles.

Consistent with the authors’ data sharing agreement, the ML predictions were confined to include only PA stream reaches. Site-wise ΣEARs were classified into three categories according to the PFAS bioeffect potential. Building upon previous research that investigated various EAR levels, a threshold of 10^−3^ was established for “Greater” PFAS bioeffect potential (Corsi et al. [13]). This threshold was determined to align closely with the prioritization of chemicals derived from established water quality guidance (Corsi et al. [35]; Alvarez et al. [44]; Oliver et al. [45]). To make three balanced categories, which is a common practice for determining ML classifications (Han et al. [46]; Alpaydin [47]), the ΣEAR threshold of <0.00002 was chosen for the “None to less” PFAS bioeffect potential. The three categories of PFAS bioeffect potential are detailed in Table 1, and site-specific categories are displayed in Figure 1 and detailed in Table S5.

### 2.3. Machine Learning Models and Feature Importance Analysis

#### 2.3.1. CNN Architecture

Recently, CNNs have been successfully implemented for ecological and water quality predictions (Pyo et al. [21]; Gandhimathi et al. [22]; Pu et al. [23]). In this study, the CNN is designed to process predictors to help classify the PFAS bioeffect potential. The network consists of multiple layers that extract and interpret features from the predictors, leading to more accurate classifications of the PFAS bioeffect potential into three categories: None to less, Existent, and Greater. This design captures both simple and complex patterns, ensuring reliable predictions across different levels of predictors.

#### Feature Extraction via Convolutional Layers

The feature extraction process in the CNN is carried out through a series of convolutional layers, each playing a critical role in enhancing the network’s performance (LeCun et al. [48]; Goodfellow et al. [49]; Gu et al. [50]). The first convolutional layer acts as a set of filters that are learned during model training, detecting fundamental patterns related to geologic, industrial, chemical, land-cover, and climatic characteristics (Krizhevsky et al. [51]; Mayr et al. [52]). These filters allow the network to capture localized, direct relationships within the data, which are essential for understanding basic patterns in PFAS bioeffect potential (LeCun et al. [48]; O’Shea and Nash [53]).

The second convolutional layer builds on these initial patterns, enabling the network to detect more complex interactions between the predictors and the classified bioeffect potentials (Goodfellow et al. [49]; Mayr et al. [52]). By analyzing larger segments of the input sequence, the second layer captures intricate dependencies, such as the interaction between land-use patterns and PFAS, or the synergistic effects of climatic and urban land-use factors (Xu et al. [54]; Rashid et al. [55]). This dual-layer structure enables the model to abstract simple patterns into higher-level features that are crucial for accurately assessing PFAS bioeffect potential (Xu et al. [54]; Li et al. [56]; Wu et al. [57]).

Furthermore, by applying this CNN structure to tabular data, we follow recent advancements that demonstrate how such models can be adapted to high-dimensional, non-image datasets by leveraging convolutional layers to model correlations between features, even without a spatial grid (Han et al. [58]). This is crucial in PFAS research, where the predictors come from diverse sources with potential complex interactions that benefit from deep learning models capable of handling such intricacies (Table 2).

#### Activation, Pooling Mechanisms, Dense Layers, and Regularization

Following feature extraction, the Rectified Linear Unit (ReLU) activation function introduces non-linearity, enabling the model to learn complex patterns. MaxPooling layers reduce dimensionality, retaining significant features and providing translation invariance, which is crucial to recognize patterns and features of geospatial predictor data regardless of their spatial variability, noise, and inconsistency. The flattened output is then processed through dense layers, where the first layer derives high-level insights, and the second layer refines these insights for accurate classification into the three PFAS bioeffect potential labels.

#### Handling Highly Correlated Variables

In CNNs, managing highly correlated variables is sometimes necessary for maintaining robustness and accuracy, especially in this study’s geospatial data, that includes potentially dozens of correlated predictors. Although CNNs inherently mitigate some correlations through their hierarchical structure and feature extraction, additional strategies that were applied herein can further enhance the model’s performance.

- L1 and L2 Regularization: L1 regularization encourages sparsity by driving some weights to zero, reducing the influence of less important, correlated features. L2 regularization penalizes large weights, ensuring the model doesn’t overly rely on any single feature. Together, these regularization techniques create a more balanced model that generalizes better to new data.
- Dropout layers: randomly deactivate neurons during training to prevent overfitting and reduce dependency on specific, potentially correlated features. This promotes generalization by diversifying the model’s learning pathways.

#### Summary of CNN Architecture

**Figure 2:**
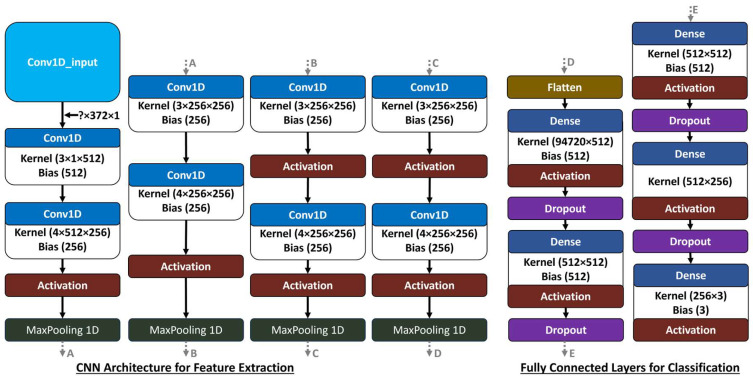
Schematic representation of the Convolutional Neural Network (CNN) architecture that illustrates each layer’s configuration, including the number of filters, kernel sizes, activation functions, and pooling layers. The architecture begins with an input layer (Conv1D_input in light blue), followed by a series of Conv1D layers (blue) with specific kernel dimensions and filter counts, each activated (red) by non-linear functions to facilitate complex feature extraction. MaxPooling1D layers (green) are interspersed throughout to downsample spatial dimensions while retaining essential features. Toward the output, fully connected Dense layers (dark blue) are utilized, incorporating dropout regularization (purple) to reduce overfitting. [The letters A through E in the CNN schematic are used to indicate the sequence of layers in the network. They don’t represent any specific meaning or functionality within the network; the ? from the Conv1D_input indicates that the batch size is dynamic and can be any positive integer].

The CNN architecture is robust and adaptable, capturing complex patterns in the geospatial predictors for classifying PFAS bioeffect potential. A CNN architecture summary is presented in Table 3 and a schematic providing an in-depth view of the CNN structure that highlights the thoughtful design choices for effective feature learning and classification is detailed in Figure 2.

#### 2.3.2. Traditional Machine Learning Models

In addition to the CNN, we trained and evaluated several traditional ML models to establish a comprehensive baseline for comparison. These models were selected based on their widespread use for environmental PFAS predictions (Vieira et al. [59]; Nguyen et al. [60]; Friedman [61]; Liu et al. [62]; Shin et al. [63]; McMahon et al. [16]) and proven efficacy in various classification tasks and included:

- Logistic Regression: A linear model (Cox [64]) that is highly valued for its simplicity and interpretability. It was implemented using the LogisticRegression class from Scikit-learn, configured to run for up to 1000 iterations to ensure convergence.
- Support Vector Machine (SVM): The SVM model (Cortes and Vapnik [65]) was configured with probability estimates enabled, which allowed for the calculation of the AUC-ROC scores—a metric that represents the model’s ability to distinguish between different classes. The use of a radial basis function (RBF) kernel was particularly important for capturing non-linear relationships within the data, making SVM an effective tool for handling complex datasets.
- Gradient Boosting: Implemented using the GradientBoostingClassifier (Friedman [61]), this ensemble method builds a series of decision trees, where each subsequent tree aims to correct the errors made by the previous ones. Gradient Boosting is known for its robustness in handling various data complexities.
- Random Forest: Another ensemble method, the Random Forest classifier (Ho [66]), constructs multiple decision trees during training and makes predictions based on the majority vote of these trees. Random Forest is particularly robust against overfitting due to its ensemble nature, which averages out the biases of individual trees.

#### 2.3.3. Model Training

The first step in our methodology was the careful preparation of the dataset, which is foundational to any successful machine learning endeavor. We divided the data into distinct training and validation sets, allocating 90% of the data for training and reserving the remaining 10% as an independent validation set. The decision to use 10% for validation was necessary due to the smaller sample size commonly encountered in environmental PFAS studies, allowing us to maximize the amount of data available for training while retaining a sufficient portion for model evaluation. This split was carried out using a stratified sampling approach, ensuring that the proportion of each class of PFAS bioeffect potential was maintained consistently across both subsets. This is especially important in classification tasks where class imbalance can lead to biased model predictions and poor generalization.

The CNN model was trained in Python version 3.11 (Python Software Foundation [67]) using Keras (Sarkar et al. [68]), a high-level neural networks API running on TensorFlow (Abadi et al. [69]). Early stopping was implemented with a patience of 20 epochs to monitor the model’s performance on the validation set during training, halting the process if the validation loss did not improve within that window. This helped prevent overfitting, where a model may perform well on the training data but poorly on unseen (out-of-sample) test data. Additionally, a maximum of 100 training epochs was set to balance performance and computation time. Model checkpointing was used to save the model’s weights at the point of highest validation accuracy.

Dropout layers with a rate of 0.50 were included to further prevent overfitting by randomly deactivating neurons during training, promoting generalization across different samples. L1 and L2 regularization with values of 1 × 10^−4^ were applied to limit over-reliance on specific features and improve the model’s robustness. These regularization techniques help control model complexity, ensuring that the network generalizes well to new data.

To ensure robustness and reliability, we conducted 25 iterations of training and testing for each model. This iterative approach mitigates the influence of random variations in model performance that can occur due to differences in data splits or initialization of model parameters. By averaging the results across multiple iterations, a more accurate and stable estimate of each model’s true performance was obtained. In Figure 1, the training and validation sites plotted represent the model having the best accuracy score (89%).

To improve our CNN’s predictive accuracy, we used Bayesian Optimization to fine-tune key hyperparameters (Head et al. [70]). This process refined filter sizes, kernel dimensions, dropout rates, learning rates, and batch sizes, leading to a model with optimal generalization and computational efficiency. For instance, the first convolutional layer was tuned with filters ranging from 32 to 1024, while kernel sizes of 2, 3, and 4 were explored to capture diverse patterns of PFAS bioeffect potential. Dropout rates (0.1 to 0.5) helped prevent overfitting, while learning rates (0.0001 to 0.01) ensured stable convergence. The batch size, set between 16 and 128, balanced memory use and training stability, and fully connected layers, ranging from 1 to 4, integrated higher-level features.

The final CNN configuration applied included:

- 3 Convolutional Stacks: with 512 filters in the first layer and 256 filters in subsequent layers.
- 3 Fully Connected Layers: with 512 and 256 neurons in the first and second layers, respectively.
- Other Hyperparameters: Kernel Sizes of 3 and 4, a Pooling Layer Size of 2, a Dropout Rate of 0.5, and a Batch Size of 6.

These hyperparameters helped to achieved improved validation accuracy, precision, recall, and F1 scores, balancing model complexity and generalization to accurately classify the PFAS bioeffect potential.

To optimize the classical models, such as Logistic Regression, SVM, Gradient Boosting, and Random Forest, we used Grid Search to systematically explore and select the best hyperparameter configurations. The final optimal values were:

- Logistic Regression: C = 1.0 (C is the inverse of the regularization strength), Penalty = L2
- SVM: C = 10 (C is the regularization that controls the trade-off between maximizing margin and minimizing classification error), Kernel = RBF
- Gradient Boosting: n_estimators = 200, Learning Rate = 0.1, max_depth = 3
- Random Forest: n_estimators = 300, max_depth = 20, min_samples_split = 5

While these tuned configurations improved each model’s performance, they also allowed for a robust comparison with the CNN. Performance metrics (Table S6) and confusion matrices (Figure S3) further illustrate each model’s classification results.

#### 2.3.4. Feature Importance Using SHAP (SHapley Additive exPlanations)

SHAP (SHapley Additive exPlanations) (Lundberg and Lee [71]) is a unified framework for interpreting the output of machine learning models. Developed from cooperative game theory, SHAP assigns an importance value, known as a SHAP value, to each feature based on its contribution to the model’s prediction.

In a cooperative game, players work together to achieve a common goal, and the Shapley value provides a fair distribution of the total payoff among the players, based on their individual contributions. In machine learning, the “players” are the features, and the “payoff” is the model’s prediction. Unlike traditional feature importance methods, SHAP provides a consistent and theoretically grounded approach that can be applied across different types of models (Lundberg et al. [72]; Molnar [73]; Lundberg and Lee [71]; Liu et al. [62]).

SHAP evaluates the contribution of each feature by considering all possible combinations of features (known as coalitions). For each coalition, the model is evaluated with and without the feature in question, and the difference in predictions is attributed to the feature’s contribution. The SHAP value for a feature is then calculated as the weighted average of these contributions across all possible coalitions.

In this study, we utilized SHAP’s Gradient Explainer, which is an extension of Integrated Gradients (Sundararajan et al. [74]) that provides approximate SHAP values. The Gradient Explainer leverages model gradients to approximate feature contributions. This approach, supported by independent theory, is particularly useful for deep learning models similar to CNNs, where understanding complex interactions between features is critical (Lundberg et al. [72]). The Gradient Explainer is described in SHAP documentation as providing approximate SHAP values, balancing computational efficiency and interpretability. SHAP enhances the interpretability of the CNN model for studying PFAS exposure and its potential effect on aquatic organisms, providing both global and local insights.

By using Gradient Explainer, we obtained both:

- Global interpretability to assess the overall importance of features across the dataset, identifying key drivers of PFAS bioeffect potential, such as land use or geologic patterns, which can inform broader land management strategies.
- Local interpretability provides insights into individual predictions, highlighting how specific factors, such as rainfall intensity or nearby industrial activity, contribute to PFAS bioeffect potential in specific streams, which can inform targeted sampling efforts.

## 3. Results

### 3.1. PFAS Concentrations

The detection of one or more PFAS occurred at 81% of the 280 stream reaches. The 19 detected PFAS included nine perfluoroalkyl carboxylates (PFCAs), five perfluoroalkane sulfonates (PFSAs), and five compounds classified as a precursor, replacement, or other chemical (Table 4). The five most frequently detected compounds in streams included PFOA (detection frequency (DF) = 75%), PFHxA (DF = 67%), PFOS (DF = 59%), perfluoropentanoate (PFPeA; DF = 57%), and PFBS (DF = 55%). Throughout all sites, these five PFAS were observed at >50% of streams; however, all other individual PFAS were observed at <50% of streams and had median concentrations that were below detection. Interquartile ranges of individual detected substance concentrations ranged from 0 (non-detect) to 5.1 ng/L, and maximum concentrations ranged from 0.6 to 120 ng/L, with 6:2 fluorotelomer sulfonate (6:2 FTS) having the maximum concentration. The median ΣPFAS concentration was 10.1 ng/L and ranged from non-detect to 268 ng/L (Table 4 and Table S1).

### 3.2. Site-Wise PFAS Bioeffects Potential

**Figure 3:**
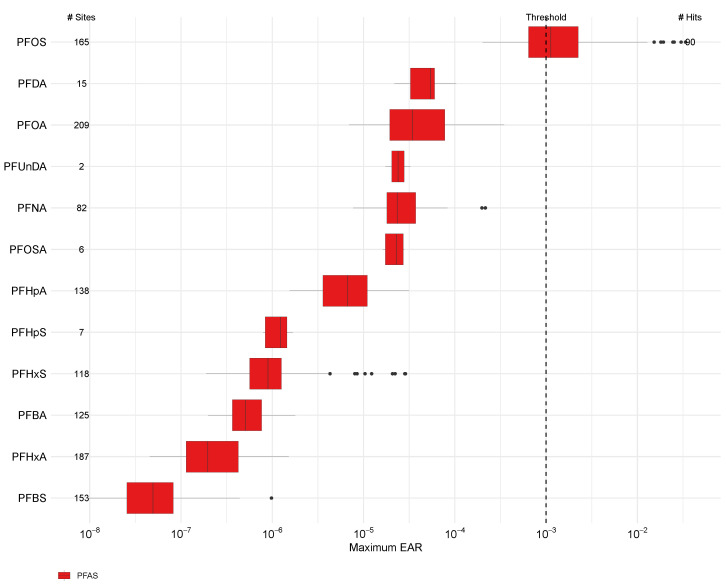
Maximum EARs for PFAS detected in 280 northeast U.S. streams, 2016 through 2023 using effect concentrations derived from ToxCast activity concentrations. The dashed line represents a threshold of 0.001 for ToxCast activity concentrations. Censored values were not included in boxplots. The number of sites (# sites) where each compound was detected is included along the y-axis. Site-wise EARs are detailed in Table S8. [Boxes, 25th to 75th percentiles; dark line, median; whiskers, data within 1.5× the interquartile range (IQR); circles, values outside 1.5× the IQR].

Throughout the combined training and validation sites, of the 19 PFAS detected, 12 had one or more ToxCast assays and measurable effects within the range of PFAS concentrations (Figure 3). The sulfonated compound PFOS had greater EAR values than any other compounds represented in ToxCast and was the only compound to exceed the EAR threshold (0.001), which occurred at 55% of the streams where it was detected. Several carboxylated compounds (PFDA, PFOA, PFUnDA, and PFNA) and one precursor (PFOSA) had median EAR values ranging from 10^−5^ to 10^−4^. The six other compounds with measurable effects (PFHpA, perfluoroheptane sulfonate (PFHpS), PFHxS, PFBA, PFHxA, and PFBS), in comparison, contributed less to the ΣEAR (median EARs < 10^−5^) (Figure S1).

In addition to individual compound EARs, a breakdown of the maximum EARs by ToxCast endpoint are presented in Figure S2. Eighteen ToxCast endpoints had measurable effects (each individual PFAS with measurable effects had one or more assays). The assay that examines the ability of the test chemical to inhibit cytochrome P450 (CYP) enzymes had greater EAR values than any other endpoints and exceeded the “Greater” EAR threshold (0.001) at 74 sites. These enzymes are crucial for metabolizing various substances, including toxins, environmental pollutants, and endogenous compounds (e.g., lipids). The phosphatase and protease were the only other ToxCast endpoints to exceed the EAR threshold at ten and three sites, respectively. Notably, the organism-level assay for zebrafish (Danio rerio) had measurable effects at 59% of stream reaches and at several sites it nearly approached an EAR of 0.001 (Figure S2 and Table S8).

**Figure 4:**
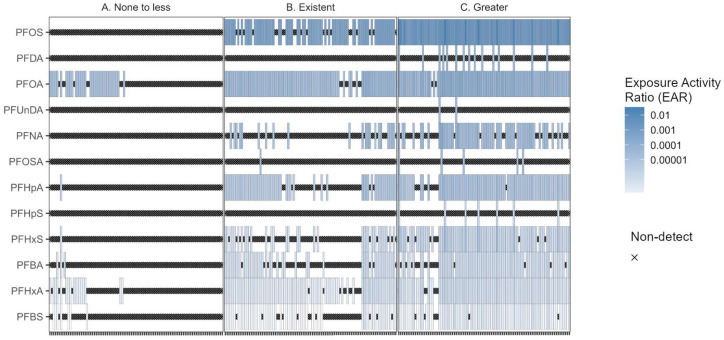
Heatmaps of maximum EARs from surface water concentrations of PFAS at 280 northeast U.S. stream reaches, 2016 through 2023 using effect concentration estimates from ToxCast activity concentrations. The x-axis represents individual sites that are separated into panels of machine learning classifications of PFAS bioeffect potential of (**A**) None to less, (**B**) Existent, and (**C**) Greater.

The site-specific maximum ΣEAR ranged from 0 to 0.034 (median = 5 × 10^−4^) (Tables S3 and S5). The training and validation sites were separated into the three classifications of PFAS bioeffect potential as described in Section 2.2. and displayed in Figure 4 and Figure S1.

Chemical analysis for combined ToxCast endpoints indicates that one-third of sites had ΣEAR for PFAS exceeding 10^−3^. Overall, the “Greater” PFAS bioeffect stream reaches had detections of all 12 PFAS with measurable effects, the “Existent” had detections of 9 PFAS, and the “None to less” had detections of 6 PFAS, but at less than half of stream reaches. While PFOS was the predominant contributor to the ΣEAR at stream reaches with “Existent” and “Greater” effects, PFOA had the highest frequency of occurrence throughout all site classifications. Throughout the 219 stream reaches that had a measurable ΣEAR, the presence of PFOS alone contributed roughly 95% to the ΣEAR.

### 3.3. Machine Learning Model Performances

The performances of five different classification models, including CNN, Logistic Regression, SVM, Gradient Boosting, and Random Forest, were evaluated over the 25 training and validation iterations using a set of key metrics, including accuracy, precision, recall, F1 score (accounts for precision and recall), and AUC-ROC (Table S6).

**Figure 5:**
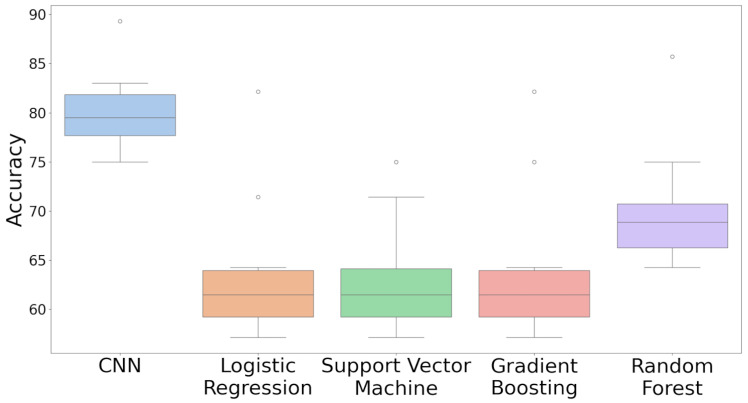
Boxplots of the accuracy (percentage) distributions across multiple iterations for each model displaying central tendency, spread, and outliers.

The median accuracy observed across 25 iterations was 79% for CNN, 68% for Random Forest, and 64% for Gradient Boosting, Logistic Regression, and SVM. The recorded accuracy varied across each iteration, with the CNN ranging from 75% to 89%, Random Forest from 64% to 86%, both Gradient Boosting and Logistic Regression from 57% to 82%, and SVM from 57% to 85% (Figure 5).

The ranges observed for each model illustrate the extent of variability in accuracy outcomes across the repeated iterations. For instance, the CNN’s range of 75% to 89% indicates the lowest and highest accuracy achieved during CNN testing, respectively. In comparison, the Random Forest, Gradient Boosting, Logistic Regression, and SVM exhibited wider ranges of accuracy. These ranges, alongside the medians, provide a comprehensive summary of the accuracy distributions, reflecting the behavior of each model under different data splits.

**Figure 6:**
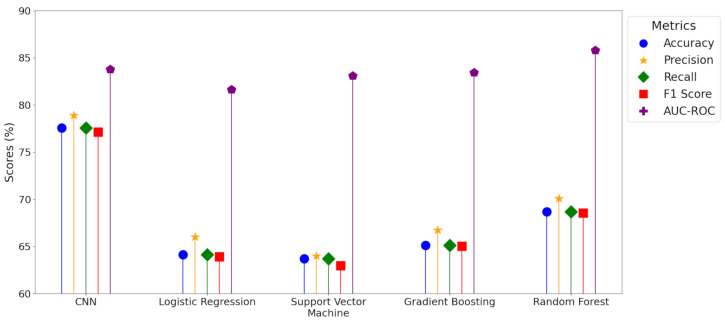
Performance trends of key metrics for machine learning models tested across 25 training and validation iterations.

Performance metrics were analyzed for each model across multiple iterations and provide detailed insights into the performance and variability of each model. Figure 6 displays mean performance metrics and Table S6 details comprehensive statistical descriptors, such as the mean, standard deviation, minimum, 25th percentile, median, 75th percentile, and maximum values for each metric.

The CNN model achieved a mean accuracy of 78%, Random Forest reached 69%, Logistic Regression and SVM both had 64%, and Gradient Boosting demonstrated 65%. For mean precision, the CNN model displayed 79% (range: 76% to 91%), Random Forest achieved 70% (range: 62% to 86%), and Logistic Regression, SVM, and Gradient Boosting had mean precision values of 66%, 64%, and 67%, respectively.

The CNN model achieved a mean recall of 78%, while Random Forest demonstrated 69%, Logistic Regression and SVM both had 64%, and Gradient Boosting recorded 65%. For mean F1 score, the CNN model attained 77% (range: 73% to 89%) and Random Forest had a mean of 69% (range: 61% to 85%). Logistic Regression, SVM, and Gradient Boosting had mean F1 scores of 64%, 63%, and 65%, respectively. In terms of mean AUC-ROC, the CNN model reached 84%, Random Forest achieved 86%, Logistic Regression had 82%, and both SVM and Gradient Boosting had 83%.

Overall, the mean values for accuracy, precision, recall, and F1 score were highest for the CNN, with the lowest corresponding standard deviations, as detailed in Table S6. Gradient Boosting had maximum standard deviations across these four performance metrics. For the AUC-ROC metric, the Random Forest model recorded the highest mean of 86%.

### 3.4. CNN Predictions of PFAS Bioeffect Potential and SHAP Feature Importance

**Figure 7:**
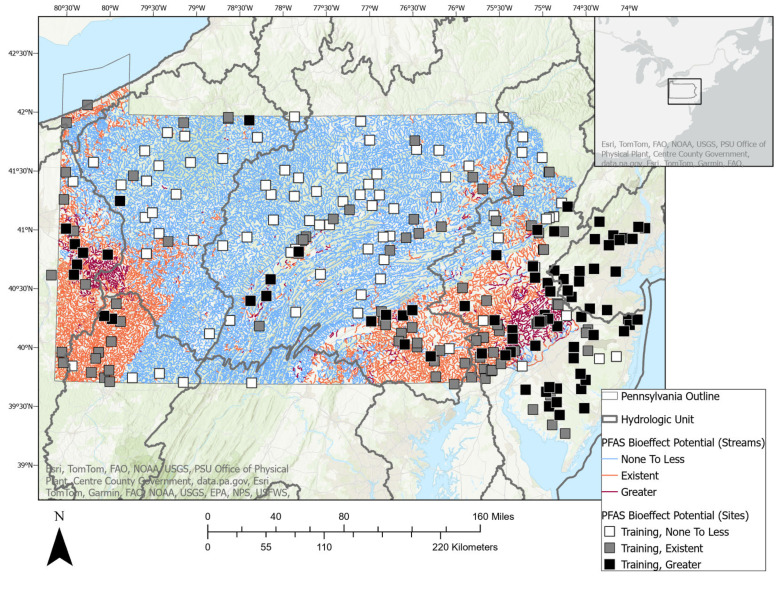
Map of the predicted PFAS bioeffect potential in NHDplus v.2.1 stream reaches of Pennsylvania. Machine learning training sites are overlaid as symbology points.

Due to its high scores across key metrics, the CNN was chosen to predict the PFAS bioeffect potential into each PA stream reach. The predictions classified 71% of PA stream reaches (ComIDs) as having “No to less” PFAS bioeffect, 24% as having “Existent” bioeffect, and 5% as having “Greater” bioeffect potential (Figure 7 and Table S9). Similar to Figure 1, the training sites plotted represent the CNN model with the highest accuracy score (89%) out of 25 iterations.

In this study, SHAP values were calculated for the predictors to determine their importance in classifying PFAS bioeffect potential. SHAP can identify specific feature interactions, revealing how the influence of one feature can vary depending on the values of others. Although SHAP has the ability to indicate whether a feature’s contribution to a prediction is positive or negative, interpretation is more nuanced because the effect can change based on the feature’s value in the context of other features and for each observational prediction. Thus, the SHAP results provided are absolute values, not relative measures, and do not indicate direction. Table 5 summarizes the SHAP values for the top 10 features having the highest contributions to the model, highlighting the most influential predictors associated with the PFAS bioeffect potential. For a full list of 56 predictors that were in at least 15 out of 25 cross-validation iterations, refer to Table S10.

SHAP identified several key attributes contributing to PFAS bioeffect potential that fall into four major categories including geological, land-use, environmental, and water resource factors. Geological factors including sinkhole density in karst landscapes, residual surficial carbonates, and percentage of sand in soil had some of the highest mean importance scores, but showed up less frequently in the SHAP cross validation. Hydrologic factors were top predictors that showed up the most frequently throughout the 25 SHAP iterations and in addition to rain event intensity, included wet deposition ammonia from manure and freshwater withdrawal. Other key features included non-alfalfa hay crop cover, commercial/service areas, high urban interfaces with high population density, and industrial and military land cover that can encompass manufacturing, landfill, and water management features (Table 5).

## 4. Discussion

### 4.1. In-Stream PFAS Concentrations and Exposure Activity Ratios

Although PFOA and PFHxA were the most frequently detected PFAS and exhibited higher median concentrations than PFOS, PFOS emerged as the primary contributor to the PFAS bioeffect potential. A recent Great Lakes surface water study similarly identified PFOS as the most prevalent PFAS contributing to ΣEAR, with aqueous concentrations that exceeded relevant effects levels (Corsi et al. [13]). The combined presence of PFOS and PFOA significantly contributed to the overall PFAS bioeffect potential, potentially due in-part to the availability of more ToxCast assays for these compounds, indicative of global regulatory concerns by numerous agencies worldwide (Corsi et al. [13]).

Several studies, including those of fish, have demonstrated that PFOS exposure can lead to differential expression of biotransformation genes, including CYP, suggesting a complex mechanism of toxicity. Furthermore, the authors indicated disruptions in lipid metabolism, with altered expression of genes associated with lipid synthesis and oxidation, leading to abnormal lipid accumulation (Corsi et al. [13]; Mihaljevic et al. [75]). CYP is expressed in fish as a stress response to help them cope and adapt to detrimental environmental conditions (Geslin and Auperin [76]). Corsi et al. [13] revealed that a cell-free assay measuring the inhibition of CYP 2C9 activity (Kavlock et al. [77]) exhibited the highest ΣEAR values in U.S. Great Lakes surface waters, primarily attributed to PFOS. Likewise, our study that utilizes the same ToxCast assays, indicates PFOS and the assay related to lipid metabolism was a significant contributor to the overall PFAS bioeffect potential (Bylund et al. [78]).

### 4.2. Machine Learning

#### 4.2.1. Comparative Model Metric Implications

The CNN’s high scores across most metrics highlights its ability to manage the intricate and high-dimensional data commonly encountered in geospatial predictors. Its ability to consistently balance precision and recall, indicated in its high F1 score, makes it particularly suited for tasks where both accuracy and the trade-off between false positives and false negatives are critical. Random Forest demonstrates proficiency in class distinction. However, CNN’s ability to consistently achieve high scores for all performance metrics (>73%) across different thresholds, in comparison to Random Forest (>60%), underscores its reliability in classifying the PFAS bioeffect potential.

The results show that Logistic Regression and SVM were not as effective in capturing complex patterns within geospatial predictors, with their mean performance scores across all five metrics ranking lowest. Although kernel-based SVMs, such as those using a radial basis function (RBF), offer non-linear boundaries and were explored, these models did not match the accuracy achieved by CNN and Random Forest in this study, likely due to the high-dimensional relationships in the data.

Gradient Boosting was moderately effective in capturing complex patterns within geospatial predictors, with its mean performance scores ranking higher than SVM and Logistic Regression, but lower than CNN and Random Forest. However, Gradient Boosting’s highest standard deviation across accuracy, precision, recall, and F1 (mean = 10%) indicated variability across multiple iterations and its performance being influenced by factors such as hyperparameter tuning and data quality (Probst et al. [79]; Chen and Guestrin [80]). Although AUC-ROC scores were lower for Gradient Boosting compared to CNN, in previous modeling of PFAS occurrences in U.S. groundwater Gradient Boosting methods have demonstrated strong testing accuracies (around 80%) and high sensitivity and specificity (McMahon et al. [16]; Tokranov et al. [81]). This success in binary classification tasks potentially indicates that Gradient Boosting may perform reliably with simpler classification targets, but may exhibit reduced accuracy when handling more nuanced tasks, such as the three-category PFAS bioeffect potential classification in PA streams.

Traditional models like Logistic Regression and Gradient Boosting can be used efficiently and interpreted easily, especially for straightforward relationships. Because they provide valuable insights into PFAS contamination and effectively link predictors such as land use and proximity to PFAS sources (McMahon et al. [16]; Tokranov et al. [81]). These models perform robustly with fewer features, making them suitable for scenarios where computational simplicity and quick interpretability are preferred.

Although robust performance can be achieved with fewer features, identifying the most valuable geospatial predictors from a vast array of options can be time-consuming. Traditional models may not capture the complex, interacting geospatial patterns inherent in PA’s diverse landscapes, which can affect predictive accuracy and generalizability. CNNs, by automatically extracting hierarchical features, are capable of handling the complexities in spatial patterns that relate to PFAS bioeffect potential.

In this study, traditional models served as benchmarks, achieving accuracies of around 65% compared to CNN’s 78%. These accuracy results demonstrate the capability of CNN to process complex datasets. However, traditional models can be effective when used in ensemble approaches, such as in Extreme Gradient Boosting or Quantitative Structure-Property Relationship (QSPR) models, as exemplified by Tokranov et al. [81] and Kowalska et al. [19], respectively.

#### 4.2.2. CNN and SHAP Implications

Our findings align with previous research, suggesting a strong association between PFAS bioeffects, as measured by ΣEAR from concentrations in environmental waters, and high urban interfaces characterized by greater wastewater effluent contributions (Barber et al. [82]; McMahon et al. [16]; Corsi et al. [13]; Smalling et al. [83]). Industrial and military land cover, another key predictor of PFAS bioeffect potential, encompasses water management features such as wastewater treatment plants. A recent study also implicated water pollution control facilities as major PFAS sources (Breitmeyer et al. [29]). Because of the strong association between wastewater treatment plants and PFAS contamination, others have proposed that improved wastewater treatment could mitigate the biological impact of PFAS in aquatic environments (Corsi et al. [13]).

Although sewage treatment plant density was included in the CNN model, it was not an important feature and may have been overshadowed by other factors identified in the SHAP analysis. These features included high urban interfaces and industrial and military land cover, which represent a combination of point and non-point PFAS sources. Consistent with our findings, PA stream reaches with military bases were recently documented to represent more than 70% of the downstream PFSA load, even when they only account for 19% of the land area (Woodward et al. [84]). Numerous studies documenting surface water PFAS contamination have identified similar key sources. For instance, industrial and military facilities, such as manufacturing plants, landfills, and military training areas, have been linked to PFAS contamination (Breitmeyer et al. [29]; Viticoski et al. [85]; McMahon et al. [16]; Imbrigiotta and Fiore [86]).

Rain event intensity emerged as a critical factor, appearing in all 25 iterations of the SHAP analysis. Although drought conditions can concentrate PFAS in surface waters due to increased evapostransportation, flooding and heavy rainfall can dilute existing concentrations but also introduce new PFAS from various sources, such as runoff from contaminated land (Kolpin et al. [87]; Kurwadkar et al. [88]). In addition, high rainfall intensity often results in combined sewer overflows, which have previously been associated to stream PFAS contamination (Breitmeyer et al. [29]). Moreover, rainwater itself can contain PFAS that can be transported to surface water through stormwater runoff (Pfotenhauer et al. [89]; Pike et al. [90]; Martinez et al. [91]). Freshwater withdrawals were also associated with the PFAS bioeffect potential. A dominant fraction of freshwater withdrawals in PA are used for drinking water and energy and occur near urban areas, which are characterized by elevated surface water PFAS concentrations (Pennsylvania Department of Environmental Protection [92]; Breitmeyer et al. [29]).

To the best of the authors’ knowledge, wet deposition ammonia from manure and non-alfalfa hay crop cover has not been explicitly identified as a direct PFAS source before. However, SHAP can capture interaction effects between features, indicating these features might indirectly influence PFAS bioeffect potential through interactions with other factors. For instance, these crops could be acting as a PFAS sink, absorbing PFAS from the soil (Adu et al. [93]). Alternatively, they might contribute to PFAS loading in streams during intense rainfall, potentially due to runoff associated with PFAS-contaminated pesticide or biosolid application (Johnson [94]; Pepper et al. [95]; Caniglia et al. [96]). Others have reported elevated PFAS in Northeastern U.S. hay crops (University of Massachusetts Amherst [97]), indicating that non-alfalfa hay crops potentially influence the environmental fate and transport of PFAS by acting as a contaminant sink.

Additionally, sinkholes that are often in karst landscapes characterized by residual carbonates, have the potential to facilitate groundwater flow and contaminant leaching. Sinkholes have been linked to surface water PFAS in PA, the U.S., and internationally (Breitmeyer et al. [29]). Although sinkholes had the highest SHAP importance score, their high uncertainty and limited appearance in SHAP (occurring in 16/25 iterations) suggest potential variability in influence, which may be due to the complex relation of sinkhole formation, residual carbonates, excessive rainfall, and aging infrastructure (White et al. [98]). There are high sinkhole densities in the Cumberland Valley and central PA, which are likely playing a role in the greater estimates of PFAS bioeffect potential in central PA stream reaches (Jones et al. [38]).

This study provides strong evidence supporting the connection between PFAS bioeffect potential and various anthropogenic factors and mixed-cover landscapes, including high urban interfaces, industrial/military land cover, agriculture, and karst topography. These findings could be used to inform comprehensive strategies to better understand and mitigate PFAS contamination and help protect aquatic ecosystems.

### 4.3. Limitations and Future Direction

To address study limitations and enhance our understanding of PFAS bioeffects potential, further research is warranted. In this study, the ΣEAR solely focuses on PFAS, neglecting the potential additive effects of other unmeasured aqueous concentrations of organic and inorganic toxic contaminants. Furthermore, the ΣEAR PFAS estimates, incorporating up to 14 PFAS, underestimate potential mixture effects due to the limited subset of PFAS monitored and having toxicity assays in the EPA ToxCast database. If further research confirms the toxic effects of PFHxS and other understudied PFAS, these compounds could be incorporated into watershed management and the CNN model (Corsi et al. [13]) and the CNN model. This study focused on PFAS exposure to aquatic biota in stream surface waters; however, the potential for additive or synergestic effects with other contaminants, such as microplastics, pharmaceuticals, and pesticides, remains a significant concern.

The absence of microplastic and respective chemical make-up concentration data for streams precluded a comprehensive assessment of their potential bioeffect. Integrating microplastics into this analysis would have posed several technical challenges. ToxCast, the toxicological assessment tool employed in this study, relies on specific chemical identifiers (CASRNs) and established toxicological benchmarks. Because microplastics exhibit diverse compositions, sizes, and surface properties, deriving standardized benchmarks for their toxicity is complex. Microplastics sorb PFAS and other contaminants that can be ingested by aquatic organisms (Alimi et al. [99]). However, certain plastics, such as those composed of polyethylene, exhibit a greater sorption capacity than other plastic types (Alimi et al. [99]). Furthermore, the potential for additive or synergistic effects between PFAS and microplastics is not fully understood, and current modeling frameworks may not adequately capture these interactions (Santhanam et al. [100]). Given the rapid evolution in this field, standardized toxicity benchmarks for microplastics are still lacking (Sarkar et al. [101]).

Other chemical contaminants, including pharmaceuticals and pesticides, may contribute to aqautic biological effects and are likely to be present in many of the watersheds studied. The co-occurrence of different chemical classes highlights the potential for interacting biological effects due to chemical mixtures, which were not represented in the ΣEAR that estimates additive effects (Corsi et al. [35]; Bradley et al. [12]; Baldwin et al. [102]).

Given the importance of high urban interfaces, industrial and military land cover, hay crop cover, and rain event intensity, future research of PFAS bioeffect potential could focus on incorporating urban-, industrial-, and agricultural-runoff-specific contaminants. Despite limitations associated with the inclusion of microplastics, future research could also prioritize the simultaneous assessment of PFAS and microplastic co-contamination. For example, advanced analytical techniques and emerging toxicological models, such as QSPR, have recently been successful in the prediction of PFAS bioconcentration factors in fish (Kowalska et al. [19]) and could potentially enable a more nuanced understanding of the combined effects of microplastic-specific chemical contaminants and PFAS on aquatic ecosystems (Alimi et al. [99]; Meng et al. [103]). Such models that correlate chemical structure with properties or activity, when combined with CNN models, could predict the effects of non-target PFAS in streams. However, non-target compound concentrations would still be necessary to calculate a comprehensive PFAS bioeffect potential based on ΣEAR. By addressing these knowledge gaps, such approaches could verify chemical co-occurrence, estimate bioeffects from multiple contaminant classes, aid in source identification, and inform effective risk mitigation strategies (Corsi et al. [13]).

Validating the CNN model through biological effect assessments of aquatic organisms could further strengthen its predictive power. ToxCast is primarily designed for assessing the toxicity of chemicals in aqueous solutions. Although there are methods to estimate potential exposure from other media (e.g., using partition coefficients for sediment concentrations), there are high uncertainties related to these estimations. In addition, PFAS concentration data for these media are not commonly available in most PA streams that were included in this analysis. Therefore, the current assessment does not account for the potential effects of PFAS associated with foam, suspended solids, or bed sediment (Interstate Technology and Regulatory Council [104]; Schwichtenberg et al. [105]). However, the primary purpose of this assessment is to provide a method for estimating the relative potential for PFAS bioeffects and prioritize sites for biological sample collection that have a range of likelihoods of biological outcomes. To meet the critical need to understand PFAS bioeffects in PA streams, the authors have estimated the potential biological effect to the best of our ability considering the existing data. Future research could consider these additional exposure pathways, as further data become available, to provide a more comprehensive assessment of PFAS risks.

The limited availability of PFAS data for larger validation and test sets necessitates further field and laboratory studies to validate the biological effects at both unaffected and PFAS-contaminated sites. The authors encountered difficulties in incorporating additional PFAS sites or a test on unseen data due to the lack of comparable PFAS measurements in other data (i.e., our ToxCast analysis included 14 compounds, but a potential unseen dataset only measured half of those). As more toxicity testing and stream concentrations become available, they could be further integrated into the CNN to enhance the model’s ability to predict potential effects and screen for uncertainty. Incorporating a larger data set with more streams into the CNN model is expected to enhance performance by improving generalization and representation learning and reducing overfitting. Computational constraints and the potential benefits of data augmentation should be evaluated to optimize the training process. Additionally, sediment and other aqueous environmental PFAS samples targeting stream segments impacted by specific industries and practices could also be integrated into the CNN to enhance predictions.

## 5. Conclusions

We optimized a CNN in a novel application for predicting stream surface water PFAS bioeffect potential and achieved a validation accuracy of roughly 78%. In comparison, the Logistic Regression and Gradient Boosting models achieved accuracies of ~65% across the same dataset. Feature importance analysis highlighted key variables that contributed to the CNN’s predictive power. The multifaceted nature of the most influential features highlights the complex interplay of diverse factors contributing to PFAS contamination in surface waters. Industrial/military and urban land cover, agricultural factors, rain event intensity, karst landscapes and sinkholes, and their interactions emerged as key determinants. These features highlight the critical yet diverse environmental and anthropogenic factors influencing PFAS contamination. However, no single feature was overwhelmingly important in the prediction process, indicating a well-balanced model where multiple features contribute to predictions. Furthermore, various factors that contribute to the occurrence of the compounds having greater biological health effects (e.g., PFOS) likely also influence the potential ΣPFAS exposure risk.

Study results provide insights for water quality and biotic monitoring efforts emphasizing the significance and strength of using advanced ML techniques to address pressing environmental issues, such as surface water PFAS contamination. By analyzing and presenting the results with state-of-the-art models like CNN, researchers can enhance their predictive capabilities at unmonitored locations and determine which geospatial features may influence water quality contaminants. Results of this study are critical to prioritize a biotic sampling scheme that aims to measure in-situ health effects, guide policy and fish sampling programs, evaluate stream water-impairments from PFAS, and communicate the ecological risks to aquatic biota associated with PFAS exposure.

## Figures and Tables

**Table 1:** Prediction categories of the per- and polyfluoroalkyl substances (PFAS) bioeffect potential (determined by the sum of Exposure Activity Ratios (ΣEAR)) utilized as the machine learning classifier.

PFAS Bioeffect Potential	ΣEAR Range	Training Site Count	Validation Site Count
None to less	<0.00002	84	10
Existent	0.00002–0.001	84	9
Greater	0.001–0.04	84	9

**Table 2:** Comparison of single vs. dual convolutional layers.

Layer Aspect	Single Convolutional Layer	Dual Convolutional Layers
Feature depth	Captures basic patterns	Detects more complex, higher-level patterns
Receptive field	Limited to small portions	Expands to cover larger portions of input
Feature abstraction	Tied closely to raw input	Produces abstract, high-level representations
Learning capacity	Limited due to fewer parameters	Increased, allowing modeling of complex relationships

**Table 3:** Summary of convolutional neural network (CNN) architecture.

Component	Configuration	Role	Significance
Input Layer	1D input vector	Prepares predictors for analysis	Maintains predictor data order, crucial for detecting patterns
First Convolutional Layer	Filters, ReLU activation	Extracts basic patterns, such as geologic and climatic characteristics	Captures fundamental geospatial signatures
Second Convolutional Layer	Filters, ReLU activation	Detects complex patterns	Identifies complex relationships between predictor variables
MaxPooling Layers	Pooling size	Reduces data dimensionality, retaining significant features	Focuses on critical features, reduces noise
First Dense Layer	Neurons, ReLU activation	Refines features into high-level representations	Synthesizes patterns into a cohesive understanding
Second Dense Layer	Neurons, ReLU activation	Enhances classification accuracy	Ensures accurate and nuanced classifications
Dropout Layers	Dropout rate	Prevents overfitting	Ensures generalization across diverse conditions
Final Output Layer	3 neurons, softmax activation	Outputs classification into impact labels	Provides clear, interpretable classifications

**Table 4:** Summary of maximum PFAS concentrations and detection frequencies in the 280 streams included in the machine learning training and validation datasets. For reporting level ranges and full chemical names refer to Table S7. nd, non-detect.

Chemical	Chemical Group	Detection Frequency (DF)	Concentration (Range) Median, ng/L	Concentration Interquartile Range, ng/L	ToxCAST Bioassay(s) Available
ΣPFAS	na	81%	(nd–268) 10.1	31.8	No
PFOA	PFCA	75%	(nd–25.0) 1.7	4.0	Yes
PFHxA	PFCA	67%	(nd–20.0) 1.5	3.9	Yes
PFOS	PFSA	59%	(nd–84.0) 1.1	3.3	Yes
PFPeA	PFCA	57%	(nd–29.0) 1.8	5.1	Yes
PFBS	PFSA	55%	(nd–53.6) 0.9	3.1	Yes
PFHpA	PFCA	49%	(nd–9.6) nd	2.0	Yes
PFBA	PFCA	45%	(nd–17.0) nd	4.4	Yes
PFHxS	PFSA	42%	(nd–61.0) nd	1.6	Yes
PFNA	PFCA	29%	(nd–12.0) nd	0.8	Yes
6:2 FTS	Precursor/Other	9%	(nd–120) nd	0.0	Yes
PFPeS	PFSA	6%	(nd–9.4) nd	0.0	No
PFDA	PFCA	5%	(nd–1.9) nd	0.0	Yes
PFHpS	PFSA	3%	(nd–1.9) nd	0.0	Yes
PFOSA	Precursor/Other	2%	(nd–1.2) nd	0.0	Yes
PFUnDA	PFCA	0.01	(nd–1.5) nd	0.0	Yes
HFPO-DA	Precursor/Other	1%	(nd–5.9) nd	0.0	No
FPePA	PFCA	<1%	(nd–34.7) nd	0.0	No
N-EtFOSAA	Precursor/Other	<1%	(nd–0.6) nd	0.0	No
N-MeFOSAA	Precursor/Other	<1%	(nd–1.6) nd	0.0	No

**Table 5:** Summary of feature importance and descriptions. Count represents the number of times the predictor was in the 25 iterations of SHapley Additive exPlanations (SHAP) output.

Predictor	Count	Importance Score (Mean ± Std. Dev)	Description
Wet Deposition Ammonia from Manure	25	0.014 ± 0.007	The fraction of the total ammonia wet deposition due to emissions from animal manure
Rain Event Intensity	25	0.013 ± 0.007	Annual average (1981–2010) of daily intensity of precipitation for a rain event where there are consecutive days with precipitation ≥ 1 mm
Freshwater Withdrawals	24	0.013 ± 0.007	County-level estimates of freshwater withdrawals from 1995–2000
Non-Alfalfa Hay	22	0.013 ± 0.009	Any type of hay crop that is not alfalfa. Can include grasses, legumes, and forbs
Industrial/Military	21	0.012 ± 0.008	Includes heavy and light industry, seaports/ harbors, manufacturing, mills/factories, utilities, waste/recycling/ landfills, energy production, warehousing/distribution, water-management features, major communication facilities, and military bases
Sand	20	0.013 ± 0.010	Average percent of sand in soil
Commercial/Services	19	0.012 ± 0.009	Includes retail stores, shopping centers, office buildings, commercial zones, professional services and organizations, universities, schools, hospitals, churches, prisons, police and fire stations, and so on
High Urban Interface	19	0.012 ± 0.009	Land in an urban area with a housing density > 500 or in or near an urban core area. Probable medium to high anthropogenic influence
Residual Carbonates	18	0.014 ± 0.009	Residual surficial materials developed in carbonate rocks, discontinuous or patchy in distribution
Sinkholes	16	0.020 ± 0.014	Mean sinkhole density, often found in karst landscapes characterized by limestone/dolomite bedrock that is susceptible to dissolution by water

## Data Availability

The original contributions and data presented in the study are either included in the article/Supplementary Materials or openly available in the U.S. Environmental Protection Agency Environment and Compliance History Online (ECHO) PFAS Analytics Tools at (accessed on 2 April 2024) https://echo.epa.gov/trends/pfas-tools; U.S. Geological Survey National Water Information System database at (accessed on 10 June 2024) http://dx.doi.org/10.5066/F7P55KJN; Sierra Club Moshannon Group (accessed on 26 November 2024) at <(https://www.springcreekwatershedatlas.org/post/pfas-in-the-spring-creek-and-bald-eagle-creek-watersheds)>; USGS New Jersey Water Science Center stream data downloaded from: Water Quality Portal: U.S. Geological Survey, 2024, USGS Water Quality Portal: Download water quality data at (accessed on 21 June 2024) https://www.waterqualitydata.us/. Geospatial predictor data is available in the U.S. Environmental Protection Agency Environment and Compliance History Online (ECHO) PFAS Analytics Tools at (accessed on 2 April 2024) https://echo.epa.gov/trends/pfas-tools and Wieczorek, M.E., Jackson, S.E., and Schwarz, G.E., 2018, Select Attributes for NHDPlus Version 2.1 Reach Catchments and Modified Network Routed Upstream Watersheds for the Conterminous United States (ver. 4.0, August 2023): U.S. Geological Survey data release, (accessed on 2 April 2024) https://doi.org/10.5066/F7765D7V. Further inquiries can be directed to the corresponding author.

## Supplementary Materials

The following supporting information can be downloaded at https://www.mdpi.com/article/10.3390/toxics12120921/s1: Figure S1: maximum Exposure Activity Ratios (EAR) from surface water concentrations of PFAS at 280 Pennsylvania and New Jersey streams, 2016 through 2023 using effect concentration estimates from ToxCast activity concentrations [The x-axis represents individual sites that are separated into panels of machine learning classifications of PFAS bioeffect potentials of A. None to less, B. Existent, and C. Greater. Each color of the stacked bar chart represents an individual PFAS]; Figure S2: maximum EAR by ToxCast endpoint for PFAS with exact Chemical Abstract Service (CAS) number matches and reliable concentration-response relationship and Activity Concentration at Cutoff (ACC) data in ToxCast, in Pennsylvania and New Jersey streams, 2016 through 2023, using effect concentrations derived from ToxCast activity concentrations [The dashed line represents a threshold of 0.001 for ToxCast activity concentrations. Censored values were not included in boxplots. The number of sites where each compound was detected is included along the y-axis. The zebrafish assay is for the species Danio rerio. Boxes, 25th to 75th percentiles; dark line, median; whiskers, data within 1.5× the interquartile range (IQR); circles, values outside 1.5× the IQR]; Figure S3: confusion matrices for the five machine learning models utilized to predict PFAS bioeffect potential in Pennsylvania stream reaches; Table S1: detected raw stream surface water per- and polyfluoroalkyl substances (PFAS) concentrations utilized to compute the chemical Exposure Activity Ratios (EAR) [Sample data is in the format of four digit year, two digit month, and two digit day (YYYYDDMM). For some data, sample month and days were not available. For full names of abbreviated chemicals, see Table S7. NHD comid; National Hydrography Dataset stream reach common identifiers are from McKay et al. [30]. The original data presented in this table are openly available, see the Data Availability Statement for further information]; Table S2: ToxCast assays excluded from analysis due to low quality dose-response curves based on anomalous values or lack of response [endPoint, name of the assay within the ToxCast database U.S. Environmental Protection Agency [15]]; Table S3: site-specific individual PFAS having exact ToxCast Chemical Abstract Service (CAS) number matches and reliable concentration-response relationship and Activity Concentration at Cutoff (ACC) measurable EARs (0.000001) and summed (ΣEAR) to represent the ΣPFAS concentration that induced a response in ToxCast biological assays [Assays within the ToxCast database U.S. Environmental Protection Agency [15]; USGS Site IDs are from the U.S. Geological Survey National Water Information System (U.S. Geological Survey [27])]; Table S4: geospatial predictors included in the machine learning models with descriptions, units, categorical themes, and data sources [Predictor data and metadata are from the U.S. Environmental Protection Agency Environment and Compliance History Online (ECHO) PFAS Analytic Tool (U.S. Environmental Protection Agency [26]) and Wieczorek et al. [41]]; Table S5: training and validation site-specific EAR categories included in the machine learning models [NHD comid; National Hydrography Dataset stream reach common identifiers are from McKay et al. [30]. USGS Site IDs are from the U.S. Geological Survey National Water Information System (U.S. Geological Survey [27])]; Table S6: PFAS bioeffect potential machine learning model metric summaries over 25 iterations of training and validation; Table S7: full chemical names of detected PFAS and the range of reporting levels (detection limits) at the 280 stream sites [na, not applicable; the original data presented in this table are openly available, see the Data Availability Statement for further information]; Table S8: site-specific PFAS ToxCast bioassays having exact ToxCast Chemical Abstract Service (CAS) number matches and reliable concentration-response relationship and Activity Concentration at Cutoff (ACC) measurable EARs (0.000001) and summed (ΣEAR) to represent the ΣPFAS concentration that induced a response in all of the ToxCast biological assays [Assays within the ToxCast database U.S. Environmental Protection Agency [15]; USGS Site IDs are from the U.S. Geological Survey National Water Information System (U.S. Geological Survey [27])]; Table S9: predicted PFAS bioeffect potential into each stream reach (National Hydrography Dataset version 2.1 comid) of Pennsylvania [NHD comid; National Hydrography Dataset stream reach common identifiers from McKay et al. [30]]; Table S10: feature importance determined by SHAP (SHapley Additive exPlanations) with predictor descriptions [Count represents the number of times the predictor was in the 25 iterations of SHAP output and predictors that were in at least 15 out of 25 iterations are presented [Predictor metadata from the U.S. Environmental Protection Agency Environment and Compliance History Online (ECHO) PFAS Analytic Tool (U.S. Environmental Protection Agency [26]) and Wieczorek et al. [41]].

## References

[1] De Silva A.O., Armitage J.M., Bruton T.A., Dassuncao C., Heiger-Bernays W., Hu X.C., Karrman A., Kelly B., Ng C., Robuck A. (2021). PFAS exposure pathways for humans and wildlife: A synthesis of current knowledge and key gaps in understanding. Environ. Toxicol. Chem..

[2] Sunderland E.M., Hu X.C., Dassuncao C., Tokranov A.K., Wagner C.C., Allen J.G. (2019). A review of the pathways of human exposure to poly-and perfluoroalkyl substances (PFASs) and present understanding of health effects. J. Expo. Sci. Environ. Epidemiol..

[3] Pennsylvania Fish and Boat Commission (2022). Commonwealth of Pennsylvania Public Health Advisory 2022 Fish Consumption. https://www.pa.gov/agencies/dep/programs-and-services/water/clean-water/water-quality/fishconsumption-advisories.html.

[4] U.S. Environmental Protection Agency (2024). Per- and Polyfluoroalkyl Substances (PFAS)|US EPA. https://www.epa.gov/sdwa/and-polyfluoroalkyl-substances-pfas.

[5] Ruffle B., Archer C., Vosnakis K., Butler J.D., Davis C.W., Goldsworthy B., Parkman R., Key T.A. (2024). US and international per- and polyfluoroalkyl substances surface water quality criteria: A review of the status, challenges, and implications for use in chemical management and risk assessment. Integr. Environ. Assess. Manag..

[6] Banyoi S.M., Porseryd T., Larsson J., Grahn M., Dinnétz P. (2022). The effects of exposure to environmentally relevant PFAS concentrations for aquatic organisms at different consumer trophic levels: Systematic review and meta-analyses. Environ. Pollut..

[7] Hamed M., Vats A., Lim I.E., Sapkota B., Abdelmoneim A. (2024). Effects of developmental exposure to individual and combined PFAS on development and behavioral stress responses in larval zebrafish. Environ. Pollut..

[8] Olker J.H., Elonen C.M., Pilli A., Anderson A., Kinziger B., Erickson S., Skopinski M., Pomplun A., LaLone C.A., Russom C.L. (2022). The ECOTOXicology Knowledgebase: A Curated Database of Ecologically Relevant Toxicity Tests to Support Environmental Research and Risk Assessment. Environ. Toxicol. Chem..

[9] Stackpoole S.M., Shoda M.E., Medalie L., Stone W.W. (2021). Pesticides in US Rivers: Regional differences in use, occurrence, and environmental toxicity, 2013 to 2017. Sci. Total Environ..

[10] Shoda M.E., Sprague L.A., Murphy J.C., Riskin M.L. (2019). Water-quality trends in U.S. rivers, 2002 to 2012: Relations to levels of concern. Sci. Total Environ..

[11] Dix D.J., Houck K.A., Martin M.T., Richard A.M., Setzer R.W., Kavlock R.J. (2006). The ToxCast Program for Prioritizing Toxicity Testing of Environmental Chemicals. Toxicol. Sci..

[12] Bradley P.M., Romanok K.M., Smalling K.L., Masoner J.R., Kolpin D.W., Gordon S.E. (2023). Predicted aquatic exposure effects from a national urban stormwater study. Environ. Sci. Water Res. Technol..

[13] Corsi S., Loken L., Ankley G., Alvarez D., Villeneuve D. (2025). Potential for biological effects of PFAS in Great Lakes tributaries and associations with land cover and wastewater effluent. Environ. Toxicol. Chem..

[14] DeCicco L., Corsi S., Villeneuve D., Blackwell B., Ankley G. (2024). toxEval: Exploring Biological Relevance of Environmental Chemistry Observations. R Package Available at CRAN. R Package Version 1.3.2. https://CRAN.R-project.org/package=toxEval.

[15] U.S. Environmental Protection Agency (2022). ToxCast & Tox21 Summary Files from Invitrodb v3.5. https://www.epa.gov/chemical-research/toxicity-forecaster-toxcasttm-data.

[16] McMahon P.B., Tokranov A.K., Bexfield L.M., Lindsey B.D., Johnson T.D., Lombard M.A., Watson E. (2022). Perfluoroalkyl and Polyfluoroalkyl Substances in Groundwater Used as a Source of Drinking Water in the Eastern United States. Environ. Sci. Technol..

[17] Dong X., Zhang Y., Wang J., Li M., Wang X., Wang Y. (2023). Prediction of 35 Target Per- and Polyfluoroalkyl Substances (PFASs) in California Groundwater Using Multilabel Semisupervised Machine Learning. Environ. Sci. Technol..

[18] DeLuca N.M., Mullikin A., Brumm P., Rappold A.G., Cohen Hubal E. (2023). Using geospatial data and random forest to predict PFAS contamination in fish tissue in the Columbia river basin, United States. Environ. Sci. Technol..

[19] Kowalska D., Sosnowska A., Zdybel S., Stepnik M., Puzyn T. (2024). Predicting bioconcentration factors (BCFs) for per-and polyfluoroalkyl substances (PFAS). Chemosphere.

[20] Khaki S., Wang L. (2019). Crop Yield Prediction Using Deep Neural Networks. Front. Plant Sci..

[21] Pyo J., Park L.J., Pachepsky Y., Baek S.S., Kim K., Cho K.H. (2020). Using convolutional neural network for predicting cyanobacteria concentrations in river water. Water Res..

[22] Gandhimathi G., Chellaswamy C., Selvan T. (2024). Comprehensive river water quality monitoring using convolutional neural networks and gated recurrent units: A case study along the Vaigai River. J. Environ. Manag..

[23] Pu F., Ding C., Chao Z., Yu Y., Xu X. (2019). Water-quality classification of inland lakes using landsat8 images by convolutional neural networks. Remote Sens..

[24] Limbu S., Glasgow E., Block T., Dakshanamurthy S. (2024). A Machine-Learning-Driven Pathophysiology-Based New Approach Method for the Dose-Dependent Assessment of Hazardous Chemical Mixtures and Experimental Validations. Toxics.

[25] Feinstein J., Sivaraman G., Picel K., Peters B., Vázquez-Mayagoitia Á., Ramanathan A., MacDonell M., Foster I., Yan E. (2021). Uncertainty-Informed Deep Transfer Learning of Perfluoroalkyl and Polyfluoroalkyl Substance Toxicity. J. Chem. Inf. Model..

[26] U.S. Environmental Protection Agency (2024). Enforcement and Compliance History Online (ECHO) PFAS Analytic Tools. https://echo.epa.gov/trends/pfas-tools.

[27] U.S. Geological Survey (2024). USGS Water Data for the Nation: U.S. Geological Survey National Water Information System Database.

[28] Roberts D. (2024). The Sprink Creek Watersehd Atlas, PFAS Survey Data. https://www.springcreekwatershedatlas.org/post/pfas-in-the-spring-creek-and-bald-eagle-creek-watersheds.

[29] Breitmeyer S.E., Williams A.M., Duris J.W., Eicholtz L.W., Shull D.R., Wertz T.A., Woodward E.E. (2023). Per- and polyfluorinated alkyl substances (PFAS) in Pennsylvania surface waters: A statewide assessment, associated sources, and land-use relations. Sci. Total Environ..

[30] McKay L., Bondelid T., Dewald T., Johnston J., Moore R., Rea A.U.S. (2012). Geological Survey NHDPlusV2 User Guide. https://www.epa.gov/waterdata/nhdplus-national-hydrography-dataset-plus.

[31] R Core Team (2024). R: A Language and Environment for Statistical Computing. https://www.R-project.org/.

[32] Blackwell B.R., Ankley G.T., Corsi S.R., DeCicco L.A., Houck K.A., Judson R.S., Li S., Martin M.T., Murphy E., Schroeder A.L. (2017). An “EAR” on environmental surveillance and monitoring: A case study on the use of exposure–activity ratios (EARs) to prioritize sites, chemicals, and bioactivities of concern in Great Lakes waters. Environ. Sci. Technol..

[33] Filer D.L., Kothiya P., Setzer R.W., Judson R.S., Martin M.T. (2016). tcpl: The ToxCast pipeline for high-throughput screening data. Bioinformatics.

[34] Fay K.A., Villeneuve D.L., Swintek J., Edwards S.W., Nelms M.D., Blackwell B.R., Ankley G.T. (2018). Differentiating pathway-specific from nonspecific effects in high-throughput toxicity data: A foundation for prioritizing adverse outcome pathway development. Toxicol. Sci..

[35] Corsi S.R., De Cicco L.A., Villeneuve D.L., Blackwell B.R., Fay K.A., Ankley G.T., Baldwin A.K. (2019). Prioritizing chemicals of ecological concern in Great Lakes tributaries using high-throughput screening data and adverse outcome pathways. Sci. Total Environ..

[36] Judson R., Richard A., Dix D.J., Houck K., Martin M., Kavlock R., Dellarco V., Henry T., Holderman T., Sayre P. (2009). The Toxicity Data Landscape for Environmental Chemicals. Environ. Health Perspect..

[37] U.S. Geological Survey (2021). Watershed Boundary Dataset (WBD). https://prd-tnm.s3.amazonaws.com/index.html?prefix=StagedProducts/Hydrography/WBD/National/.

[38] Jones J., Doctor D., Wood N., Falgout J., Rapstine N. (2021). Closed Depression Density in Karst Regions of the Conterminous United States: Features and Grid Data.

[39] Blodgett D., Johnson M. (2023). nhdplusTools: Tools for Accessing and Working with the NHDPlus.

[40] Wieferich D., Gressler B., Krause K., Wieczorek M., McDonald S. (2022). xstrm local.

[41] Wieczorek M., Jackson S., Schwarz G. (2018). Select Attributes for NHDPlus Version 2.1 Reach Catchments and Modified Network Routed Upstream Watersheds for the Conterminous United States (Ver. 4.0, August 2023).

[42] Peterson R.A., Cavanaugh J.E. (2020). Ordered quantile normalization: A semiparametric transformation built for the cross-validation era. J. Appl. Stat..

[43] U.S. Environmental Protection Agency (2010). Level III and IV Ecoregions of the Continental United States. U.S. EPA Office of Research & Development (ORD)—National Health and Environmental Effects Research Laboratory (NHEERL). https://www.epa.gov/eco-research/level-iii-and-iv-ecoregions-continental-united-states.

[44] Alvarez D.A., Corsi S.R., De Cicco L.A., Villeneuve D.L., Baldwin A.K. (2021). Identifying chemicals and mixtures of potential biological concern detected in passive samplers from Great Lakes tributaries using high-throughput data and biological pathways. Environ. Toxicol. Chem..

[45] Oliver S.K., Corsi S.R., Baldwin A.K., Nott M.A., Ankley G.T., Blackwell B.R., Villeneuve D.L., Hladik M.L., Kolpin D.W., Loken L. (2023). Pesticide prioritization by potential biological effects in tributaries of the Laurentian Great Lakes. Environ. Toxicol. Chem..

[46] Han J., Kamber M., Pei J. (2011). Data Mining: Concepts and Techniques.

[47] Alpaydin E. (2020). Introduction to Machine Learning.

[48] LeCun Y., Bengio Y., Hinton G. (2015). Deep learning. Nature.

[49] Goodfellow I., Bengio Y., Courville A. (2016). Deep Learning.

[50] Gu J., Wang Z., Kuen J., Ma B., Shahroudy A., Shuai B., Liu T., Wang X., Wang G., Chen J. (2018). Recent Advances in Convolutional Neural Networks. Pattern Recognit..

[51] Krizhevsky A., Sutskever I., Hinton G.E. ImageNet Classification with Deep Convolutional Neural Networks. Proceedings of the Advances in Neural Information Processing Systems.

[52] Mayr A., Klambauer G., Unterthiner T., Hochreiter S. (2016). DeepTox: Toxicity Prediction Using Deep Learning. Front. Environ. Sci..

[53] O’Shea K., Nash R. (2015). An Introduction to Convolutional Neural Networks. arXiv.

[54] Xu K., Ba J., Kiros R., Cho K., Courville A., Salakhutdinov R., Zemel R., Bengio Y. Show, Attend and Tell: Neural Image Caption Generation with Visual Attention. Proceedings of the 32nd International Conference on Machine Learning (ICML).

[55] Rashid R., Ahmed K., Anwar W., Ali H. (2019). XTox: Toxicity Prediction Using Shallow Learning Models. Comput. Chem. Eng..

[56] Li J., Monroe W., Jurafsky D. Visualizing and Understanding Neural Models in NLP. Proceedings of the 5th Workshop on Vision and Language.

[57] Wu Z., Zhang F., Pang X., Wu X., Cao W., Liu R. (2018). Convolutional Neural Networks for Toxicity Prediction. J. Chem. Inf. Model..

[58] Han H., Li Y., Zhu X. (2019). Convolutional neural network learning for generic data classification. Inf. Sci..

[59] Vieira V.M., Hoffman K., Shin H.M., Weinberg J.M., Webster T.F., Fletcher T. (2013). Perfluorooctanoic Acid Exposure and Cancer Outcomes in a Contaminated Community: A Geographic Analysis. Environ. Health Perspect..

[60] Nguyen T.V., Reinhard M., Gin K.Y.H. (2016). Sorption equilibria of perfluoroalkyl acids between sediment and water: Influence of sediment organic carbon and molecular structure. J. Hazard. Mater..

[61] Friedman J.H. (2000). Greedy Function Approximation: A Gradient Boosting Machine. Ann. Stat..

[62] Liu Y., Chen Z., Wang X. (2022). XGBoost model as an efficient machine learning approach for PFAS removal: Effects of material characteristics and operation conditions. Environ. Res..

[63] Shin H.M., Vieira V.M., Ryan P.B., Detwiler R., Sanders B., Steenland K., Bartell S.M. (2011). Environmental fate and transport modeling for perfluorooctanoic acid emitted from the Washington Works Facility in West Virginia. Environ. Sci. Technol..

[64] Cox D.R. (1958). The regression analysis of binary sequences. J. R. Stat. Soc. Ser. B (Methodol.).

[65] Cortes C., Vapnik V. (1995). Support-vector networks. Mach. Learn..

[66] Ho T.K. (1995). Random decision forests. Proceedings of the 3rd International Conference on Document Analysis and Recognition.

[67] Python Software Foundation (2023). Python 3.11. https://www.python.org/downloads/release/python-3110/.

[68] Sarkar D., Bali R., Ghosh T. (2018). Hands-On Transfer Learning with Python: Implement Advanced Deep Learning and Neural Network Models Using TensorFlow and Keras.

[69] Abadi M., Agarwal A., Barham P., Brevdo E., Chen Z., Citro C., Corrado G.S., Davis A., Dean J., Devin M. (2015). TensorFlow: Large-Scale Machine Learning on Heterogeneous Systems. http://tensorflow.org/.

[70] Head T., Cherti M., Pedregosa F., Zhdanov M., Louppe G., Raffel C., Mueller A., Fauchere N., McInnes L., Grisel O. (2018). Scikit-Optimize: Sequential Model-Based Optimization with Scikit-Learn. https://scikit-optimize.github.io/.

[71] Lundberg S.M., Lee S.I., Guyon I., Luxburg U.V., Bengio S., Wallach H., Fergus R., Vishwanathan S., Garnett R. (2017). A Unified Approach to Interpreting Model Predictions. Proceedings of the 31st International Conference on Neural Information Processing Systems, NIPS’17.

[72] Lundberg S.M., Erion G., Chen H., DeGrave A., Prutkin J.M., Nair B., Katz R., Himmelfarb J., Bansal N., Lee S.I. (2020). From Local Explanations to Global Understanding with Explainable AI for Trees. Nat. Mach. Intell..

[73] Molnar C. (2019). Interpretable Machine Learning: A Guide for Making Black Box Models Explainable.

[74] Sundararajan M., Taly A., Yan Q. Axiomatic Attribution for Deep Networks. Proceedings of the 34th International Conference on Machine Learning (ICML), PMLR.

[75] Mihaljevic I., Vujica L., Dragojavic J., Loncar J., Smital T. (2024). Differential Toxicity of Perfluorooctane Sulfonate (PFOS) in Wild-Type and Oatp1d1 Mutant Zebrafish Embryos. bioRxiv.

[76] Geslin M., Auperin B. (2004). Relationship between changes in mRNAs of the genes encoding steroidogenic acute regulatory protein and P450 cholesterol side chain cleavage in head kidney and plasma levels of cortisol in response to different kinds of acute stress in the rainbow trout (Oncorhynchus mykiss). Gen. Comp. Endocrinol..

[77] Kavlock R., Chandler K., Houck K., Hunter S., Judson R., Kleinstreuer N., Knudsen T., Martin M., Padilla S., Reif D. (2012). Update on EPA’s ToxCast program: Providing high throughput decision support tools for chemical risk management. Chem. Res. Toxicol..

[78] Bylund J., Ericsson J., Oliw E.H. (1998). Analysis of cytochrome P450 metabolites of arachidonic and linoleic acids by liquid chromatography–mass spectrometry with ion trap MS2. Anal. Biochem..

[79] Probst P., Wright M.N., Boulesteix A.L. (2019). Hyperparameters and tuning strategies for random forest. Wiley Interdiscip. Rev. Data Min. Knowl. Discov..

[80] Chen T., Guestrin C. (2016). XGBoost: A Scalable Tree Boosting System. Proceedings of the 22nd ACM SIGKDD International Conference on Knowledge Discovery and Data Mining.

[81] Tokranov A.K., Ransom K.M., Bexfield L.M., Lindsey B.D., Watson E., Dupuy D.I., Stackelberg P.E., Fram M.S., Voss S.A., Kingsbury J.A. (2024). Predictions of groundwater PFAS occurrence at drinking water supply depths in the United States. Science.

[82] Barber L.B., Keefe S.H., Brown G.K., Furlong E.T., Gray J.L., Kolpin D.W., Meyer M.T., Sandstrom M.W., Zaugg S.D. (2013). Persistence and potential effects of complex organic contaminant mixtures in wastewater-impacted streams. Environ. Sci. Technol..

[83] Smalling K.L., Romanok K.M., Bradley P.M., Morriss M.C., Gray J.L., Kanagy L.K., Gordon S.E., Williams B.M., Breitmeyer S.E., Jones D.K. (2023). Per-and polyfluoroalkyl substances (PFAS) in United States tapwater: Comparison of underserved private-well and public-supply exposures and associated health implications. Environ. Int..

[84] Woodward E.E., Senior L.A., Fleck J.A., Barber L.B., Hansen A.M., Duris J.W. (2024). Using a Time-of-Travel Sampling Approach to Quantify Per-and Polyfluoroalkyl Substances (PFAS) Stream Loading and Source Inputs in a Mixed-Source, Urban Catchment. ACS ES&T Water.

[85] Viticoski R.L., Wang D., Feltman M.A., Mulabagal V., Rogers S.R., Blersch D.M., Hayworth J.S. (2022). Spatial distribution and mass transport of Perfluoroalkyl Substances (PFAS) in surface water: A statewide evaluation of PFAS occurrence and fate in Alabama. Sci. Total Environ..

[86] Imbrigiotta T.E., Fiore A.R. (2021). Distribution of Chlorinated Volatile Organic Compounds and Per- and Polyfluoroalkyl Substances in Monitoring Wells at the Former Naval Air Warfare Center, West Trenton, New Jersey, 2014–17.

[87] Kolpin D.W., Hubbard L.E., Cwiertny D.M., Meppelink S.M., Thompson D.A., Gray J.L. (2021). A comprehensive statewide spatiotemporal stream assessment of per-and polyfluoroalkyl substances (PFAS) in an agricultural region of the United States. Environ. Sci. Technol. Lett..

[88] Kurwadkar S., Dane J., Kanel S.R., Nadagouda M.N., Cawdrey R.W., Ambade B., Struckhoff G.C., Wilkin R. (2022). Per-and polyfluoroalkyl substances in water and wastewater: A critical review of their global occurrence and distribution. Sci. Total Environ..

[89] Pfotenhauer D., Sellers E., Olson M., Praedel K., Shafer M. (2022). PFAS concentrations and deposition in precipitation: An intensive 5-month study at National Atmospheric Deposition Program–National trends sites (NADP-NTN) across Wisconsin, USA. Atmos. Environ..

[90] Pike K.A., Edmiston P.L., Morrison J.J., Faust J.A. (2021). Correlation analysis of perfluoroalkyl substances in regional US precipitation events. Water Res..

[91] Martinez B., Da Silva B.F., Aristizabal-Henao J.J., Denslow N.D., Osborne T.Z., Morrison E.S., Bianchi T.S., Bowden J.A. (2022). Increased levels of perfluorooctanesulfonic acid (PFOS) during Hurricane Dorian on the east coast of Florida. Environ. Res..

[92] Pennsylvania Department of Environmental Protection PA Water Use Annual Summary Report. Commonwealth of Pennsylvania. https://www.pa.gov/agencies/dep/data-and-tools/reports/water-reports.html.

[93] Adu O., Ma X., Sharma V.K. (2023). Bioavailability, phytotoxicity and plant uptake of per-and polyfluoroalkyl substances (PFAS): A review. J. Hazard. Mater..

[94] Johnson G.R. (2022). PFAS in soil and groundwater following historical land application of biosolids. Water Res..

[95] Pepper I.L., Brusseau M.L., Prevatt F.J., Escobar B.A. (2021). Incidence of Pfas in soil following long-term application of class B biosolids. Sci. Total Environ..

[96] Caniglia J., Snow D.D., Messer T., Bartelt-Hunt S. (2022). Extraction, analysis, and occurrence of per-and polyfluoroalkyl substances (PFAS) in wastewater and after municipal biosolids land application to determine agricultural loading. Front. Water.

[97] University of Massachusetts Amherst (2024). Manure Application on Hay Fields. Online Resource. https://ag.umass.edu/crops-dairy-livestock-equine/fact-sheets/manure-application-on-hay-fields.

[98] White E.L., Aron G., White W.B. (1986). The influence of urbanization of sinkhole development in central Pennsylvania. Environ. Geol. Water Sci..

[99] Alimi O.S., Farner Budarz J., Hernandez L.M., Tufenkji N. (2018). Microplastics and nanoplastics in aquatic environments: Aggregation, deposition, and enhanced contaminant transport. Environ. Sci. Technol..

[100] Santhanam S.D., Ramamurthy K., Priya P.S., Sudhakaran G., Guru A., Arockiaraj J. (2024). A combinational threat of micro-and nano-plastics (MNPs) as potential emerging vectors for per-and polyfluoroalkyl substances (PFAS) to human health. Environ. Monit. Assess..

[101] Sarkar S., Diab H., Thompson J. (2023). Microplastic pollution: Chemical characterization and impact on wildlife. Int. J. Environ. Res. Public Health.

[102] Baldwin A.K., Corsi S.R., De Cicco L.A., Lenaker P.L., Lutz M.A., Sullivan D.J., Richards K.D. (2016). Organic contaminants in Great Lakes tributaries: Prevalence and potential aquatic toxicity. Sci. Total Environ..

[103] Meng L., Zhou B., Liu H., Chen Y., Yuan R., Chen Z., Luo S., Chen H. (2024). Advancing toxicity studies of per-and poly-fluoroalkyl substances (pfass) through machine learning: Models, mechanisms, and future directions. Sci. Total Environ..

[104] Interstate Technology and Regulatory Council (2020). PFAS Technical and Regulatory Guidance Document and Fact Sheets PFAS. https://pfas-1.itrcweb.org/.

[105] Schwichtenberg T., Bogdan D., Carignan C.C., Reardon P., Rewerts J., Wanzek T., Field J.A. (2020). PFAS and dissolved organic carbon enrichment in surface water foams on a northern US freshwater lake. Environ. Sci. Technol..

